# Navigating Asymptomatic Mitral Regurgitation: Diagnostic Dilemmas and Treatment Strategies

**DOI:** 10.7759/cureus.61191

**Published:** 2024-05-27

**Authors:** Moiz Saeed, Rand Sabanci, Harith Ghnaima, Kevin Watat, Dina Shaban, Georgette Nader, Sandeep Banga, Matthew Wilcox

**Affiliations:** 1 Internal Medicine, Michigan State University, East Lansing, USA; 2 Internal Medicine, Michigan State University College of Human Medicine, East Lansing, USA; 3 Internal Medicine, BronxCare Health System, New York, USA; 4 Internal Medicine, Michigan State Sparrow Hospital, Lansing, USA; 5 Cardiology, Michigan State University, East Lansing, USA; 6 Cardiology, Sparrow Thoracic and Cardiovascular Institute, Lansing, USA

**Keywords:** valve surgery, valve repair, mitral valve, cardiology, guideline, mr, mitral regurgitation

## Abstract

This case report explores the complexities involved in the diagnosis and management of asymptomatic mitral regurgitation (MR) in a 64-year-old male presenting with an incidental systolic murmur. Torrential MR with flail mitral valve (MV) segments was identified through comprehensive imaging and clinical evaluation, including echocardiography and catheterization. The discussion highlights the nuances of surgical timing, emphasizing the importance of tailored approaches based on left ventricular (LV) function and dilation. This report sheds light on the evolving landscape of managing asymptomatic MR, underscoring the need for balancing surveillance with proactive intervention to optimize patient outcomes.

## Introduction

Mitral regurgitation (MR) affects approximately 10% of adults aged 75 years or older, a figure expected to rise alongside our rapidly aging population [1-3]. This condition manifests in two forms: primary MR, stemming from intrinsic mitral valve (MV) apparatus pathology, and functional MR, which arises from left ventricular (LV) disease, leading to geometric alterations in the mitral apparatus despite an anatomically normal MV.

Determining the timing of surgery in asymptomatic patients with degenerative mitral regurgitation (DMR) poses a clinical conundrum, as these patients frequently lack guideline indications for surgical intervention over extended periods. International guidelines differ in their view toward preemptive surgery for asymptomatic patients due to scarce and conflicting data. The European Society of Cardiology (ESC) advises a conservative approach involving safe monitoring until symptoms or guideline thresholds arise. In contrast, the American Heart Association/American College of Cardiology (AHA/ACC) guidelines advocate proactive surgery based on comparative studies.

Current AHA guidelines, predominantly drawn from observational studies, advocate for MV surgery with a class I recommendation in symptomatic patients with chronic severe primary MR, provided the LV dysfunction is not severe [4]. In cases of asymptomatic patients without LV dysfunction, MV surgery may be considered reasonable (class IIa recommendation) if surgical risk remains low (≤1% mortality), and there is a high likelihood (>95%) of achieving durable MV repair. Active surveillance has emerged as a viable strategy, demonstrating safety and yielding excellent outcomes, with long-term survival rates comparable to the general population [5]. However, some experts have proposed early surgical intervention in asymptomatic patients, postulating potential benefits associated with proactive management [6-8].

The advent of comprehensive echocardiographic assessment has significantly enhanced the accurate diagnosis of MR, enabling precise identification of its underlying pathophysiological mechanisms. Through selective correction of organic MV pathology, MV repair preserves both the continuity of the MV apparatus and LV systolic function. Given the clinical feasibility of predicting successful MV repair and the consistently favorable long-term outcomes associated with this approach [9,10], there is a need to engage in direct comparisons between watchful waiting strategies and early MV repair.

## Case presentation

A 64-year-old male from Michigan, with a history of migraines, presented to his primary care physician for evaluation, during which a systolic murmur was incidentally detected at his apex. Despite denying symptoms such as exertional dyspnea, orthopnea, paroxysmal nocturnal dyspnea, lower extremity swelling, palpitations, dizziness, or syncope, he reported occasional fleeting episodes of chest discomfort over the past few months. Initial laboratory investigations, including troponin and B-type natriuretic peptide (BNP), were unremarkable.

**Figure 1:**
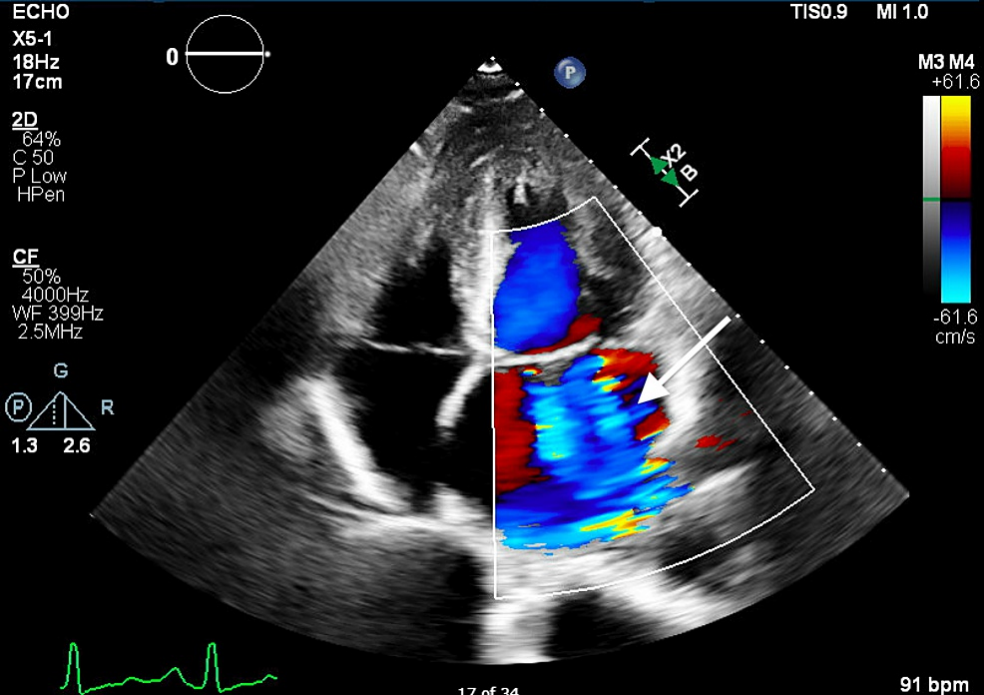
Transthoracic echocardiogram four-chamber view revealing severe torrential mitral regurgitation

**Figure 2:**
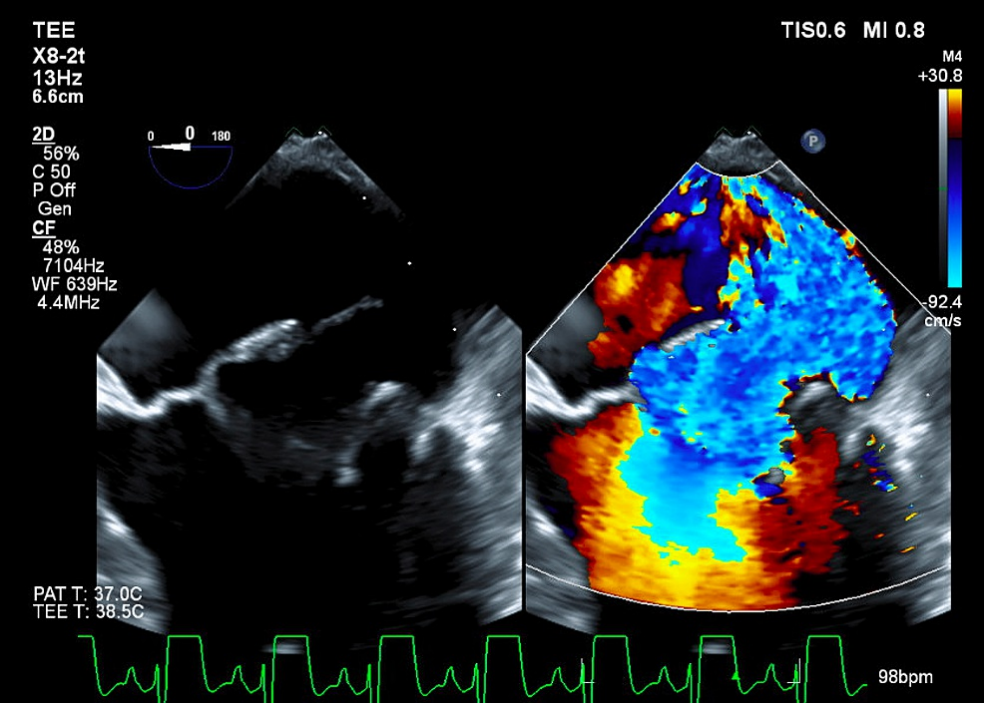
Transesophageal echocardiogram still image to better visualize severe mitral regurgitation

**Figure 3:**
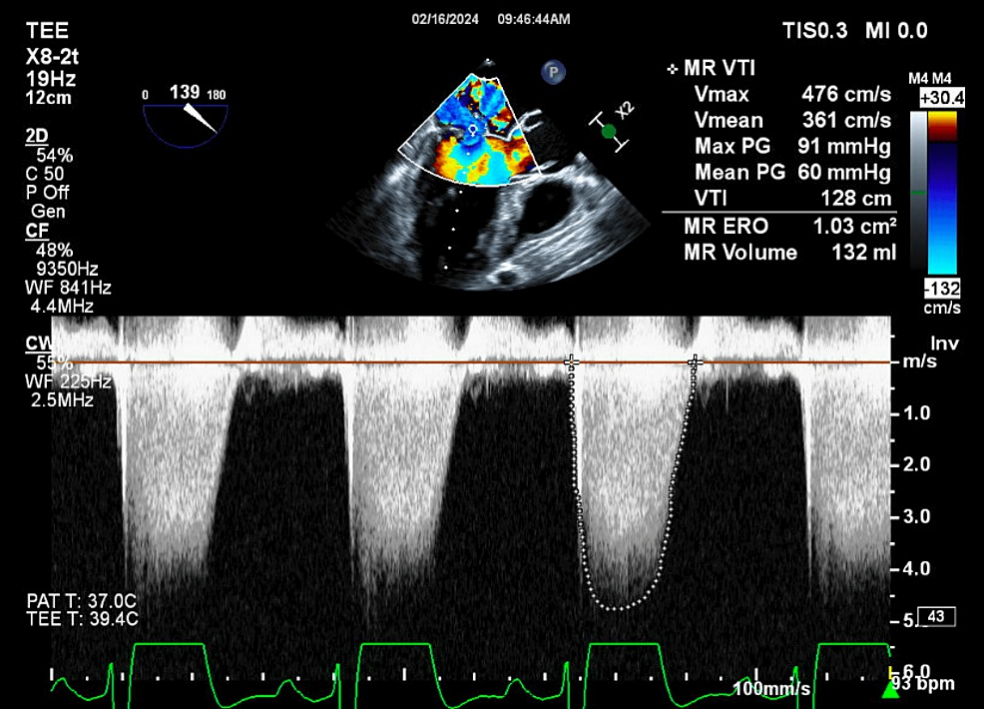
Transesophageal echocardiogram with mitral valve hemodynamics

Further assessment via transthoracic echocardiography revealed a concerning finding of torrential MR, attributed to a flail segment of the anterior leaflet (Video 1, Figure 1). Notably, his LV size was normal, with a preserved ejection fraction (EF). Subsequent evaluation in the outpatient cardiology clinic on the same day led to the performance of a transesophageal echocardiogram, which elucidated the presence of flail segments involving both A1/A3 and P2 segments of the MV leaflet. This pathology was associated with torrential eccentric, posteriorly directed MR, alongside systolic flow reversal in the right upper pulmonary vein (RUPV) and left upper pulmonary vein (LUPV) (Videos 2-3, Figures 2-3).

**Video 1:** Transthoracic echocardiogram parasternal long axis view showing flail segment of anterior mitral valve leaflet

**Video 2:** Transesophageal echocardiogram four-chamber view revealing flail mitral valve leaflet

**Video 3:** Transesophageal echocardiogram revealing severe, torrential mitral regurgitation

Following these diagnostic procedures, right and left heart catheterization demonstrated normal coronary arteries but revealed elevated left-sided pressures, including a pulmonary capillary wedge pressure (PCWP) of 19 mmHg and a mean pulmonary artery pressure (mPAP) of 24 mmHg. The patient was diagnosed with severe asymptomatic MR and, in light of these findings, he was initiated on furosemide 40 mg oral daily to address his elevated left heart pressures and promptly referred for MV surgery.

## Discussion

MR initiates compensatory mechanisms in the heart in order to maintain cardiac output. Prolonged MR can lead to eccentric hypertrophy of the cardiac myocytes and increased left ventricular end-diastolic volume (LVEDV), thereby enhancing stroke volume [11]. This adaptive phase, characterized by an enlarged left ventricle and left atrium (LA), delays symptoms of pulmonary congestion; however, sustained compensation becomes unsustainable, resulting in contractile dysfunction, further LV volume increases, reduced cardiac output, elevated LV filling pressures, and eventual symptom manifestation. While ACC/AHA guidelines recommend MV surgery for all symptomatic patients with severe MR, the management approach for asymptomatic patients is nuanced, contingent upon clinical and anatomical considerations associated with chronic severe MR [11].

The evaluation of MR involves various diagnostic methods, each offering unique insights into disease severity and prognosis. All patients with suspicion of MR should undergo echocardiography for evaluation of severity and mechanism. MR severity can be quantified by measuring the vena contracta width, regurgitant orifice area, regurgitant volume, and regurgitant fraction. Additionally, LV function, LV and LA size, and pulmonary artery pressures can also be assessed. Left ventricular ejection fraction (LVEF), although not a direct measure of myocardial function, is crucial for assessing MR severity, often underestimating LV dysfunction in MR cases. LV global longitudinal strain (LV-GLS) serves as an early indicator of LV dysfunction, aiding in the detection of subclinical myocardial dysfunction in MR. BNP levels are associated with LV dimensions, symptom development, and outcome, and hence valuable in predicting symptom onset or LV dysfunction, particularly in asymptomatic patients. Exercise testing provides an objective assessment of symptoms, functional capacity, and MR severity dynamics. Cardiac MRI (CMRI) complements echocardiography, offering excellent reproducibility and valuable data on LV volumes and function, including regurgitant volume, which can identify patients at risk for adverse outcomes, guiding surgical decision-making. A comprehensive approach employing these diagnostic methods is essential for an accurate assessment and timely intervention in patients with MR.

Surgical correction remains the cornerstone of treatment for patients with chronic severe MR, as it is the only intervention shown to alter the disease course effectively. There is a lack of evidence supporting the efficacy of medical therapy in delaying symptoms, preventing surgery, or improving patient outcomes. However, medical therapy targeting other comorbid conditions has been shown to improve outcomes. Afterload reduction in patients with MR who also have hypertension, or guideline-directed medical therapy for concomitant LV systolic dysfunction, has demonstrated efficacy in reducing the severity of MR [12].

Surgical correction for chronic severe MR in asymptomatic patients hinges largely on anticipated surgical risks and outcomes. Whenever feasible, opting for MV repair presents several advantages over replacement as it preserves the natural anatomical relationship between the valve apparatus and the papillary muscles, thereby contributing to the preservation of LV shape and function [13]. The pathology of the MV influences the decision for MV replacement compared to repair. Even in cases where valve replacement becomes necessary, surgeons may still achieve chordal preservation, offering similar benefits for LV geometry and function. Moreover, the mortality rate associated with MV repair is notably low (0-1.2%), contrasting with the significantly higher rate for valve replacement (3.8%) [11]. Of particular concern is the risk of ischemic stroke, estimated to be less than 2% for patients undergoing repair compared to 6% for those undergoing valve replacement [11].

Currently, recommendations for surgical intervention in asymptomatic patients with severe MR primarily focus on the presence of LV dysfunction (LVEF 30-60%) or dilation (LV end-systolic dimension >40 mm) [11]. These indicators suggest that cardiac remodeling is no longer able to compensate for MR and predicts functional deterioration. However, the recommendation for surgery in asymptomatic patients with severe MR without associated LV dysfunction or dilation is less straightforward. There is a class IIa indication for surgical correction in these patients if the likelihood of successful repair is high (>90% in experienced centers) [11]. Additionally, patients with other sequelae of chronic severe MR such as atrial fibrillation and pulmonary hypertension also have a class IIa indication for surgical repair [14]. However, recent studies have indicated that a significant number of asymptomatic patients with severe MR meeting ACC/AHA criteria for surgery are not referred, highlighting the need to reaffirm current guidelines among physicians caring for these patients [15].

Recent scrutiny has redirected focus towards watchful waiting due to two pivotal advancements: a high number of centers achieving MV repair rates exceeding 95% with minimal operative risk below 0.5%, and an increasing acknowledgment that delaying intervention until symptoms or ventricular dysfunction arise might heighten long-term mortality and heart failure risks, despite the option of "rescue surgery" [16,17].

Recent studies have highlighted the importance of prompt surgical intervention in patients with mitral valve regurgitation, particularly when accompanied by a flail leaflet, as it is linked to improved long-term survival and reduced risk of heart failure, even without traditional class I indications for surgery [6,11]. However, conflicting evidence still exists, with some studies suggesting that prophylactic surgery does not necessarily offer a definitive survival advantage compared to surgery triggered by clinical or echocardiographic criteria [18,19]. Despite successful outcomes reported in experienced centers, further research is still required to establish clear guidelines regarding the timing of intervention in asymptomatic patients with severe MR.

## Conclusions

Managing asymptomatic MR poses various challenges, with the timing of surgery still a matter of debate. Guidelines offer recommendations based on LV function and dilation, but individualized decisions are crucial. This case report throws light on the ongoing debate over the timing of surgical intervention in asymptomatic patients, with growing evidence favoring early surgery, especially in centers with high repair success rates and low operative risk, due to its potential benefits for long-term survival and heart failure prevention. The report also emphasizes the effectiveness of active surveillance and the need for further research to clarify guidelines and optimize management strategies for asymptomatic severe MR. Collaboration among clinicians, surgeons, and researchers is essential for effectively navigating this complexity in asymptomatic MR care.
